# Assessment of Asthma Control and Quality of Life among Asthmatic Patients Attending Armed Forces Referral and Teaching Hospital, Addis Ababa, Ethiopia

**DOI:** 10.1155/2020/5389780

**Published:** 2020-07-28

**Authors:** Tesfalidet Gebremeskel Zeru, Ephrem Engidawork, Alemseged Beyene Berha

**Affiliations:** Department of Pharmacology and Clinical Pharmacy, School of Pharmacy, College of Health Sciences, Addis Ababa University, Addis Ababa, Ethiopia

## Abstract

**Background:**

The primary goal of asthma management is to achieve good asthma control. However, poor patient-physician communication, unavailability of appropriate medications, and lack of long-term goals have made asthma control difficult in developing countries. Poor assessment of asthma control and quality of life is a major cause of suboptimal asthma treatment worldwide, and information regarding this issue is scanty in developing countries like Ethiopia. This study thus attempted to assess the level of asthma control and quality of life in asthmatic patients attending Armed Forces Referral and Teaching Hospital.

**Methods:**

A cross-sectional study comprising 184 physician-diagnosed asthmatic patients was conducted using interview, chart review, and prescription assessment. Asthma control was assessed using Asthma Control Test, while asthma quality of life was assessed using Mini-Asthma Quality of Life Questionnaire (mini-AQLQ). Spearman's rank correlation analysis was performed to understand the relationship between mean mini-AQLQ score and asthma control. Receiver operating characteristic curve analysis was performed to establish cut-off values for mini-AQLQ.

**Results:**

Asthma was uncontrolled in 67.9% subjects. There was a strong correlation between asthma control and quality of life (rs = 0.772; *P* < 0.01). A cut-off value for the quality of life was established at 4.97. Majority of the patients were taking two or three antiasthmatic drugs. Oral tablet and inhaler short-acting beta agonists (SABA) were the frequently combined drugs. Uncontrolled asthma was associated with middle-aged adults (adjusted odds ratio (AOR) = 6.31; 95% CI: 2.06, 19.3; *P* = 0.001), male gender (AOR = 0.38; 95% CI: 0.15, 0.98; *P* = 0.044), married (AOR = 0.24; 95% CI: 0.08, 0.78; *P* = 0.017), comorbidities (AOR = 0.23; 95% CI: 0.09, 0.61; *P* = 0.003), and oral SABA use (AOR = 0.22; 95% CI: 0.09, 0.59; *P* = 0.003). Male gender (AOR = 0.36; 95% CI: 0.16, 0.84; *P* = 0.018), intermittent asthma (AOR = 0.18; 95% CI: 0.04, 0.86; *P* = 0.032), use of oral corticosteroids (AOR = 0.22; 95% CI: 0.06, 0.73; *P* = 0.013), and SABA (AOR = 0.39; 95% CI: 0.17, 0.89; *P* = 0.026) were found to have a significant association with poor asthma-related quality of life.

**Conclusion:**

The findings collectively indicate asthma remains poorly controlled in a large proportion of asthma patients in the study setting. Moreover, quality of life appears to be directly related to asthma control. Healthcare providers should therefore focus on asthma education with an integrated treatment plan to improve asthma control and quality of life.

## 1. Background

Asthma is a heterogeneous disease, usually characterized by chronic airway inflammation that causes the following symptoms such as wheeze, shortness of breath, chest tightness, and/or cough and by variable expiratory airflow limitation [1]. Asthma is a problem worldwide, with an estimated 334 million affected individuals. It is the 14^th^ most important disorder in the world in terms of the extent and duration of disability. The results of phase I of the International Study of Asthma and Allergies in Childhood reported a prevalence of asthma ranging from 4.4% to 21.5% and 4.6% to 9.1% in Africa and Ethiopia, respectively [2, 3]. Moreover, for people in older age groups, premature death due to asthma contributes more to the burden of disease [4]. Unavailability of appropriate and affordable medications, poor knowledge of patients, and poor communication between physician and patient increased the burden of asthma in Ethiopia [5].

The long-term goal of asthma management is to achieve and maintain asthma control. This in turn can be achieved through a continuous cycle of treatment assessment and adjustment as well as the review of response. The level of asthma control is the extent to which the manifestations of asthma can be observed in the patient or have been reduced or removed by treatment. Asthma control has two domains: symptom control (previously called “current clinical control”) and the control of future risk of adverse outcomes [6].

Unlike other chronic diseases, such as diabetes mellitus and hypertension, the possibility of a relationship between current control and future risk of asthma adverse outcomes has received little attention by both health practitioners and patients. Exacerbations are recognized as a common clinical manifestation in asthma patients. Repeated exacerbations are, however, known to be the most important potential risk for hospitalization or death. Also, a patient with poorly controlled asthma is associated with significant impairment of quality of life (QoL) and psychosocial and economic consequences [7]. Moreover, airway remodeling is a prominent feature of asthmatic patients and caused by impaired lung development or accelerated loss of lung function [7–9].

According to the executive summary of the Global Initiative for Asthma (GINA) dissemination committee report “asthma control” is achieved when there is suppression of asthma symptoms resulting in reduction in frequency of rescue *β*2-agonist use and prevention of exacerbations [10]. Despite the availability of these guidelines that enumerate goals of asthma control and specific management strategies, asthma remains relatively poorly controlled in different age groups across the globe [11, 12]. While uncontrolled asthma substantially contributes to the burden on the healthcare system, its effects on the quality of life (QoL) of those affected should not be underestimated. As such, assessing patients' QoL constitutes an important aspect of the management of patients with asthma [13]. This requires improvement in the quality of asthma management. However, there is a knowledge gap due to limited research conducted on QoL and level of asthma control among asthmatic patients in Ethiopia. Therefore, the present study is aimed at measuring asthma-related QoL and level of asthma control as well as establishing their relationship.

Determining asthma-related QoL and the rate of control of asthma and identifying factors associated with the control, as well as reviewing patients' management and knowing the level of patients' satisfaction, would help to target those at most risk and hence reduce asthma impairment risk. Furthermore, such knowledge could help guide clinical management, drug supply management, and patient education and increase patient satisfaction.

## 2. Methods

### 2.1. Study Area

The study was conducted at the outpatient clinic of Armed Forces Referral and Teaching Hospital (AFRTH), which is one of the tertiary-level specialized referral and teaching hospitals in Addis Ababa, Ethiopia. It provides free medical services to members of the Ethiopian defense forces, their family, and civil workers at the Ministry of Defense, Ethiopia.

### 2.2. Study Design and Period

The hospital-based cross-sectional study was conducted from July 2015 to October 2015 through data abstraction format, which involved a review of medical charts and prescriptions as well as patient interview.

### 2.3. Study Population and Inclusion Criteria

Eligible patients were identified by the study team at the outpatient chest clinic of the study site. Majority of asthma patients are monitored clinically by physicians with a scheduled visit of every 4-6 weeks depending on their disease conditions. All physician-diagnosed asthmatic patients who fulfilled the inclusion criteria were included. Patients aged ≥18, on active follow-up, receiving asthma treatment for at least 6 months and willing to participate in the study, were included. Pregnant women (due to varying effects of pregnancy on bronchial asthma) and patients who had other respiratory comorbid diseases (pneumonia, COPD) and/or unstable heart failure (due to cofounding effects), had acute exacerbated asthma (to avoid an overestimated uncontrolled asthma in the study area), had missing (incomplete) data, and are not willing to participate in the study were excluded.

### 2.4. Data Collection Procedures

Data was collected by one nurse and two pharmacists. The data collectors were trained for two days on how to conduct patient interview and how to review medical charts and prescriptions. Patients were given information about the study, and a verbal informed consent was obtained before recruitment. A structured data abstraction tool was used to collect the data from medical charts and current prescriptions of surveyed patients. Collected data ranged from sociodemographic characteristics (age, sex, education level, marital status, and occupation) through disease-related factors (asthma severity, duration of diagnosis, asthma exacerbation in the last year, and comorbid condition/s) to drug-related factors (type of antiasthmatic medication/s before visit, prescription review after visit, and treatment modification).

### 2.5. Asthma Control Test (ACT) and Mini-Asthma Quality of Life Questionnaire (Mini-AQLQ)

Data were collected using pretested Asthma Control Test (ACT) and Mini-Asthma Quality of Life Questionnaire (mini-AQLQ), which were translated from English to Amharic for better understanding by the study participants, and back translation was done to check the consistency of meaning. Those questionnaires were filled out by face-to-face interview.

### 2.6. Asthma Severity Assessment

The severity of asthma was classified based on Global Initiative for Asthma (GINA) criteria [12].

### 2.7. Asthma Control Assessment

Asthma control was assessed using ACT questionnaire. The questionnaire is composed of five items: shortness of breath, nighttime waking, interference with activity, rescue bronchodilator use, and patient rating of asthma control, over the past month. Each item is scored using a 1–5 scale and then summed up (total score, 5–25). The score was then classified as well controlled (20-25), not well controlled (16-19), and very poorly controlled (5-15) asthma. For the purpose of data analysis, the total score was dichotomized. Accordingly, low and medium control scores were reassigned as uncontrolled (not well controlled and very poorly controlled) with a score of less than 20 and high control scores was regarded as controlled (well controlled) with a score of greater than or equal to 20. The ACT has been validated against a specialist's rating of control and spirometry as well as QoL [14–16].

### 2.8. Asthma QoL Assessment

Asthma QoL was measured using mini-AQLQ, a validated disease specific questionnaire [17]. This instrument is an asthma-specific questionnaire with 15 items that provides an overall summary index and assesses four domains of HRQoL: activity limitation, symptoms, emotional function, and environmental exposures during the preceding 2 weeks. The response options for each of the 15 items are on a 7-point Likert scale, ranging from 1 (totally limited) to 7 (not at all limited). The QoL score was used as a continuous variable, in which higher scores indicate no or less impairment and lower scores indicate severe impairment due to asthma.

### 2.9. Statistical Analysis

The data were checked for completeness and then entered and analyzed using SPSS software package version 21. Descriptive statistics such as frequency, percentage, mean, and standard deviation (SD) were employed to summarize study variables and evaluate distribution of responses. Spearman's rank correlation coefficients were used to study the associations between the outcome variables. Receiver operating characteristic (ROC) analysis was done with ACT score as an outcome and various mini-AQLQ cut point levels as predictors. The curve is created by plotting the true positive rate (TPR) or sensitivity against the false positive rate (FPR) at various threshold settings. Sensitivity was defined as the proportion of subjects with subsequent uncontrolled asthma who were in the high-risk group. Specificity was defined as the proportion of participants with well-controlled asthma who were not in the high-risk group. The optimal cut point of the mini-AQLQ for predicting emergency hospital care was determined by means of stepwise logistic regression analyses [18, 19]. Finally, Youden index (*J*) was calculated (*J* = sensitivity + specificity − 1). The index is defined for all points of an ROC curve, and the maximum value of the index was used as a criterion for selecting the optimum cut-off point [20].

Univariate binary logistic regression analysis was performed to relate each variable to asthma control and QoL. From the univariate analysis, those variables with *P* < 0.25 were selected for multivariable regression analysis. The multivariable regression analysis was also used to assess the predictability of the independent variables of asthma control and QoL of asthmatic patients and to estimate the odds ratios (OR), 95% confidence intervals (CI), and *P* values. The association was declared significant at *P* < 0.05.

## 3. Results

### 3.1. Sociodemographic and Disease Characteristics

Out of the 189 recruited patients, 184 patients were included in the study. Five questionnaires were rejected because of incompleteness. Almost two-third (68%) of the patients were scored less than or equal to 19, corresponding to uncontrolled asthma. The overall mean of the mini-AQLQ score in the study population out of a possible mean score of 7 was 4.497 (SD + 1.24). The highest and lowest domains of the mini-AQLQ were 4.7 (SD + 1.1) and 4.0 (SD + 1.7), for activity and environmental domains, respectively. Based on GINA severity classification, 12.5% of the participants had intermittent asthma and 87.5% had persistent asthma. About one-third of the patients had one or more comorbidities. The major comorbidities observed were cardiovascular diseases and diabetes mellitus. The characteristics of the patients are presented in Table 1.

### 3.2. Pattern of Antiasthmatic Drug Use

### 3.3. Medication Use prior to Hospital Visit

Drug utilization before visit was separately studied since self-medication in asthma is very common as the drugs are available without prescription. Treatment with antiasthmatic medication was found to involve monotherapy as well as combination therapy (Table 2). Looking at drug utilization pattern prior to patient visit revealed that multiple-drug therapy (two drugs 48.9%, three drugs 4.9%) was used by a greater number of patients as compared to single-drug therapy (46.2%). Oral and inhaler short-acting beta agonist (SABA) medications were the two most often used combination of medicines used by patients with uncontrolled asthma.

### 3.4. Medications Prescribed after Visit

Among 184 study participants, 163 had received antiasthmatic medications. The drug regimen was modified for 133 (72.3%) patients on their subsequent visit. Multiple-drug therapy was prescribed for majority of the patients (81%). The rate of ICS prescription alone or in combination with LABA was 22.8% (Table 2).

**Figure 1:**
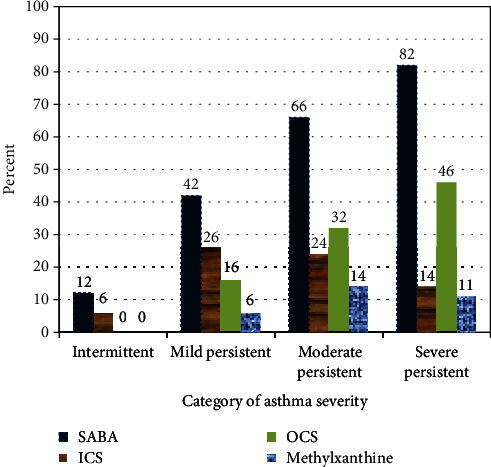
Medications prescribed after visit by type of asthma severity at Armed Forces Referral and Teaching Hospital, Addis Ababa, Ethiopia, July-October 2015 (*n* = 184).

Patients with intermittent asthma were found to inappropriately receive an ICS. Only 14% of patients with severe persistent asthma used ICS. In addition, use of quick-relief medications such as SABA was high in this group (82%) (Figure 1). Overall, drug prescription showed that oral and inhaler SABA medications were the two most often used combination of medicines. Antibiotic prescription without major findings of infection was seen in 14.7% patients with persistent asthma. Tetracyclines, macrolides, and amoxicillin were the major antibiotics utilized.

### 3.5. ACT and Mini-AQLQ Status

The mean ACT score was 14.8 (SD ± 5.6). When the cut-off for asthma control was taken as 20, 125 (67.9%) patients had uncontrolled asthma and 59 (32.1%) patients had controlled asthma. The overall mean mini-AQLQ score out of a possible mean score of 7 was 4.49 (SD ± 1.27).

### 3.6. Analysis of Relationships between Asthma Control and Quality of Life

**Figure 2:**
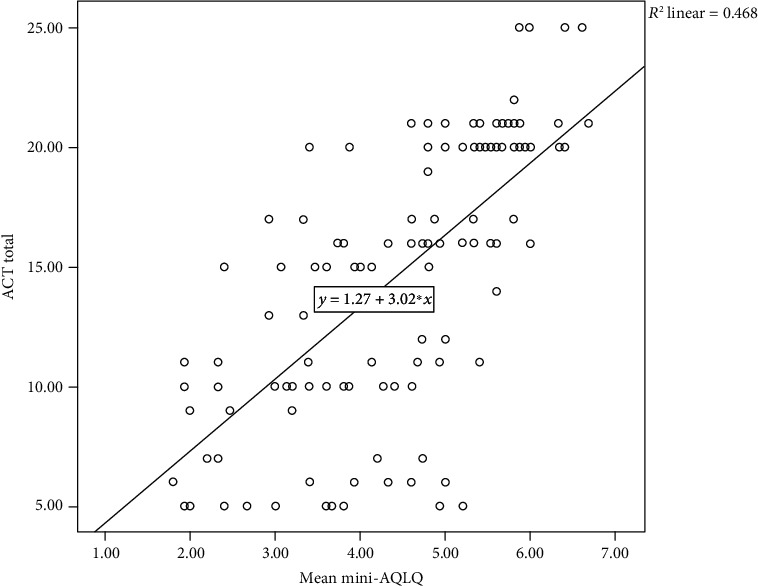
Correlation between Asthma Control Test and Mini-Asthma Quality of Life Questionnaire scores at Armed Forces Referral and Teaching Hospital, Addis Ababa, Ethiopia, July-October 2015 (*n* = 184).

Spearman's rank-order correlation analysis showed that asthma control and mean mini-AQLQ score were directly correlated (rs = 0.772; *P* < 0.01) (Figure 2). Mini-AQLQ increased with the increasing level of asthma control.

**Figure 3:**
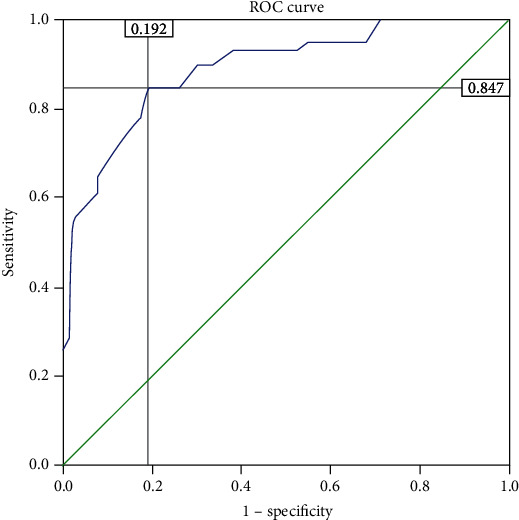
Receiver operating characteristic curve of Mini-Asthma Quality of Life Questionnaire versus Asthma Control Test at Armed Forces Referral and Teaching Hospital, Addis Ababa, Ethiopia, July-October 2015 (*n* = 184).

This study attempted to anticipate asthma control on the basis of patient QoL and to see whether the mean mini-AQLQ score could help in predicting the level of asthma control. The ROC curve was used to quantify how accurately mini-AQLQ can discriminate states of asthma control, uncontrolled representing “diseased” and controlled representing “nondiseased.” Patients were classified as controlled or uncontrolled according to ACT. The curve was created by plotting the true positive rate (sensitivity) against the false positive rate (1 − specificity) at various threshold settings (Figure 3). By calculating Youden index (*J* = sensitivity + specificity − 1) for each coordinate point of the ROC curve, the maximum value of the index was used as a criterion for selecting the optimum cut-off point. The best cut-off value that maximizes sensitivity and specificity was 4.97. At this mini-AQLQ value (4.97), the sensitivity was 0.85 and specificity was 0.81 (1–specificity = 0.19). This cut point was used to assess management effectiveness, and it was shown that while 74 patients had values > 4.97, the rest (110) had values < 4.97.

### 3.7. Association between the Outcome Variables

Among patients who had controlled asthma, almost 85% of them had good QoL. By contrast, higher percentage of the patients had poor QoL (80.8%) among those who had uncontrolled asthma. The odds of being uncontrolled were almost twentyfold higher for patients who had poor QoL compared to those who had good QoL (Table 3).

### 3.8. Factors Associated with Uncontrolled Asthma

Multivariable logistic regression analysis showed that there was no statistically significant association between duration of asthma diagnosis, use of OCS, and hospital admission in the past one year due to exacerbated asthma with uncontrolled asthma. Nevertheless, age, gender, marital status, comorbidities, and type of medication used had significant association. Those 35-64 years of age (AOR = 6.31; 95% CI: 2.06, 19.3; *P* = 0.001) were 6 times more likely to have uncontrolled asthma than those with the age category of 18-34 years. The odds for development of uncontrolled asthma in women was 62% lower than men (AOR = 0.38; 95% CI: 0.15, 0.98; *P* = 0.044). Married participants had a 24% less likelihood (AOR = 0.24; 95% CI: 0.08, 0.78; *P* = 0.017) to develop uncontrolled asthma than singles. Asthma was vastly uncontrolled in those patients diagnosed with nonrespiratory comorbidities (AOR = 0.23; 95% CI: 0.09, 0.61; *P* = 0.003). Asthma was more controlled in those patients who was not utilizing oral SABA (AOR = 0.22; 95% CI: 0.09, 0.59; *P* = 0.003) (Table 4).

### 3.9. Factors Associated with Poor Quality of Life

QoL was dichotomized, and those with mini-AQLQ scores < 4.97 were categorized as having poor asthma-related QoL. Variables associated with control status were gender, asthma severity, comorbidities, and use of oral asthma medications. The odds to have poor QoL was decreased by 64% (AOR = 0.36; 95% CI: 0.16, 0.84; *P* = 0.018) for female patients than male patients. Likewise, the odds for patients with intermittent asthma was 82% lower than those with severe persistent asthma (AOR = 0.18; 95% CI: 0.04, 0.86; *P* = 0.032). Those who did not utilize oral SABA (AOR = 0.39; 95% CI: 0.17, 0.89; *P* = 0.026) and OCS (AOR = 0.22; 95% CI: 0.06, 0.73; *P* = 0.013) had a decreased rate of poor asthma-related QoL (Table 5).

## 4. Discussion

The long-term goal of asthma management is to achieve and maintain asthma control. Controlled asthma can be achieved in a continuous cycle to assess, adjust treatment, and review response. The aim of continuous assessment is to reduce the burden to patients and their risk of exacerbations, airway damage, and medication side effects and to increase patient QoL. Assessment of QoL is relevant to clinical practice because treatment planning and progression are focused on the patient rather than on the disease.

The present study found a strong association between asthma control and asthma-related QoL. The mean asthma QoL scores were significantly higher in patients achieving good control compared to patients who did not achieve control. However, although QoL in asthma improved when asthma was controlled, optimal scores were not always observed; i.e., the achievement of asthma control did not necessarily show achievement of maximal QoL.

### 4.1. Level of Asthma Control and Determinants

Numerous studies have reported suboptimal controlled asthma, including Israel (37%) [21], Nigeria (37%) [22], Saudi Arabia (37%) [23], Cameroon (58%) [24], Italy (36.9%) [25], and the present study (32.1%) using the same tool. This difference in the level of asthma control might be due to higher use of anti-inflammatory drugs like ICS in most of the studies. In this study, only 29 subjects (15.7%) used ICS alone or in combination with LABA and such lower use of these medications is reported in other studies [22, 26] as well. Most of the patients used only medications that quickly relieve asthma symptoms, which have no role in controlling the underlying inflammation of the airways. In addition, factors that contribute for poor control of asthma, include, among others, difference in use of guidelines and implementation, poor treatment adherence, lack of patients' knowledge about the disease, and the presence of common comorbidities in asthma [27, 28].

Variables associated with control status were age, gender, marital status, comorbidities, use of oral rescue medication, and systemic corticosteroid use. The data revealed that the risk of having uncontrolled asthma is higher in adults compared to young adults. This finding is in line with studies conducted elsewhere, including Jimma, Ethiopia [29]; Saudi Arabia [23]; Turkey [30]; and the USA [31]. In contrast with previous findings [32, 33], females appear to have higher overall ACT scores compared to males. In these studies, females constitute majority of the study participants, but in the current study, the male population was higher. Higher likelihood of uncontrolled asthma in the face of the lower number in females could also be related to the site of study (military hospital). Hence, social rationalization (maybe a military woman tends to be courageous than most civilians) could also have a potential importance. However, this phenomenon needs to be further evaluated in other sociocultural setting. Asthma is often associated with various comorbidities, which may influence asthma control and response to treatment. Comorbidity showed association with asthma control, and this is in agreement with a study conducted in Portugal, where patients with one and two comorbidities had a 4.2 and 5.6 more chance, respectively, to develop uncontrolled asthma than those with no comorbidity. Increased odds of uncontrolled asthma among patients with comorbidity could be due to different reasons [25, 34]. Identification and treatment of comorbidities is now recognized as an integral part of core management of asthma particularly in the more severe forms of the disease, though the effect of treating comorbidities on asthma severity and long-term clinical outcomes remains to be seen.

### 4.2. Level of Asthma QoL and Determinants

The results show that asthma-related QoL is not optimal. From the four domains of mini-AQLQ, the environmental mean score was the lowest domain of mini-AQLQ with a mean of 4.1 (SD ± 1.7). This was in line with studies conducted elsewhere [35]. This might be because asthmatic patients tend to be affected more by environmental stimuli than other domains.

Poor QoL is indicative of asthma exacerbations. The present study revealed that about 60.3% of patients had poor QoL, and this could probably be the reason why about 24.3% of patients had been admitted to ICU or intubated in the last year.

The mini-AQLQ cut-off was obtained through ROC curve analysis by assuming better control is related to better QoL [34]. The AUC (0.89) shows a high level of sensitivity and specificity between ACT and mini-AQLQ. This was consistent with a study done in Portugal in which the AUC was 0.878 [34]. By performing Youden index (*J*), the cut-off value for the mini-AQLQ score was found to be 4.97, which serves as a commonly used measure of overall management effectiveness [36–38]. This analysis suggests that a mini-AQLQ score of <4.97 is related to worse asthma control and lower QoL, while a score of >4.97 is related to better control and higher QoL. This assertion is supported by the observation that achievement of good QoL was 7-fold higher when asthma was controlled than uncontrolled (Table 3). The cut-off value for the QoL measurement score using the mini-AQLQ was established as 5.4 in Portugal [34]. Schatz et al. [18] recommended a mini-AQLQ cut point between 3.0 and 6.0, with 0.1 increments serving as potential predictors for future exacerbation. Thus, a cut-off point 4.7 on the mini-AQLQ lies within the range and provides high sensitivity.

In this study, male gender, severe asthma, oral corticosteroid use, and oral SABA use were found to have a significant association with poor asthma QoL. Asthma severity was found to correlate with asthma QoL scores by many researchers [19, 34, 39]. It has been demonstrated that patients with severe persistent asthma have poor disease specific QoL scores in contrast to patients with intermittent asthma.

Despite guideline recommendations, patients who used oral SABA and oral OCS prior to hospital visits had poor QoL. A study done in The Netherlands found that asthma patients treated according to the guideline had significantly higher mean HRQoL score than those that are not (5.7 vs. 5.3, *P* < 0.01) [40]. In addition, Oni et al. [39] found that Nigerian patients who took nil medication, bronchodilators alone, and combination of ICS with bronchodilators had a mean value of 4.58, 4.78, and 6.09, respectively. Similarly, in the current study, patients who took ICS and LABA had the highest mini-AQLQ score.

### 4.3. Pattern of Antiasthmatic Drug Use

In this study, the pattern of drug utilization before visit showed that 40.2% of the patients had routinely used only oral SABA for the long-term control of asthma. The high dependence on SABA is supported by data from some other studies. In Nigeria, about 34% of patients were using oral SABA alone for maintenance therapy [41]. In Jimma, 58.4% of the patients used oral SABA in which the odds of uncontrolled asthma was increased by about 32.6% [29]. In the USA, uncontrolled asthma was increased by 1.7 times in patients who did not use controller medications than those who use controller medications [31]. The frequent use of SABA among asthmatics might be explained by the following: a lot of patients do not see asthma as a chronic disease, lack of understanding that asthma can be controlled with appropriate use of ICS, unavailability and unaffordability of ICS medication, or the treating physicians did not prescribe it.

This study shows that majority of the patients were taking multiple-drug therapy (two drugs 48.9%, three drugs 4.9%) prior to visit. The rate of multiple-drug therapy (53.8 vs.73.7%) and ICS prescription (15.7 vs. 22.8%) was a little bit increased. Oral and inhaler SABA medications were the two most often used combination of medicines.

After hospital visit, drug treatment was modified for 133 (72.3%) of patients. The subsequent treatment modification shows that patients were not able to control their asthma or not satisfied with drugs utilized before visiting the hospital. Since some antiasthma medications are over the counter in Ethiopia, patients purchase rescue medications (both oral and inhalation SABA) without prescription. In addition, this study also found that antibacterial medications were prescribed for asthmatic patients without major clinical, laboratory tests and radiologic supportive findings for the presence of infection. This shows that most of the patients had received antibiotics without confirming infection, and this could possibly result in over or misuse of antibiotics that culminates into development of resistance. A similar notion that antibiotics might be used (irrationally) had also been reported by Prasad et al. [42]. In connection with this, Darmon et al. [43] conducted a cross-sectional study using database in primary care of France and Italy and found 37.1% and 42.2% of antibiotic prescription, respectively. International guidelines do not recommend use of antibiotics in asthma patients unless there is a strong evidence of infection [44, 45]. Nevertheless, physicians commonly prescribe antibiotics to patients with acute asthma exacerbations. Some studies reported that oral antibiotics particularly macrolides could improve some subjective parameters, bronchial hyperresponsiveness, and airway inflammation. However, they have no benefits on lung function or overall asthma control. In fact, they have acquired wide use due to their anti-inflammatory and prokinetic properties [46]. Indeed, a randomized trial in Australia found macrolides to be an important additional therapy in refractory asthma [47].

In accordance with several studies, the reasons why physicians inappropriately prescribe antibiotics are as follows. First, for patient satisfaction or pressure, patients may expect to get a prescription at clinic visit, whether or not an antibiotic is necessary, since these drugs will make them feel better. Second, for uncertain diagnosis, physicians may go ahead and prescribe antibiotics because they perceive the risk of not prescribing them as greater than that from unnecessary antibiotic use [48–51]. Asthma patients who are prescribed with systemic corticosteroids may be more likely to receive an antibiotic [52].

## 5. Conclusion

Asthma control was very low and achieved only in under a fifth of asthmatic patients attending AFRTH, Addis Ababa, Ethiopia. QoL appears to be strongly and directly associated with asthma control. A cut-off value for the health-related QoL measurement score using the mini-AQLQ was established at 4.97, with values higher than this indicating better asthma control and QoL.

## Figures and Tables

**Table 1:** Sociodemographic and clinical characteristics of asthmatic patients at Armed Forces Referral and Teaching Hospital, Addis Ababa, Ethiopia, July-October 2015 (*n* = 184).

Sociodemographic and clinical characteristics	Number (%)
Age, mean (SD)	44.1 (±13.6)
Gender	
Male	104 (56.5)
Female	80 (43.5)
Educational status	
No formal education	28 (15.2)
Primary education	42 (22.8)
Secondary education	71 (38.6)
Higher institute	43 (23.4)
Duration of asthma^∗^	
≤14.8 years	89 (48.4)
>14.8 years	95 (51.6)
Asthma severity	
Intermittent	25 (13.6)
Mild persistent	66 (35.9)
Moderate persistent	54 (29.3)
Severe persistent	39 (21.2)
ACT	
Well controlled	59 (32.1)
Partially controlled	36 (19.6)
Very poorly controlled	89 (48.4)
Mini-AQLQ, mean (SD)	
Symptoms domain	4.49 (1.24)
Activity domain	4.59 (1.2)
Emotional domain	4.7 (1.1)
Environmental domain	4.09 (1.7)
Comorbidity	
Yes	65 (35.3)
No	119 (64.7)
Admitted to ICU or intubated in the last 12 months	
Yes	39 (21.2)
No	145 (78.8)

ACT = Asthma Control Test; mini-AQLQ = Mini-Asthma Quality of Life Questionnaire; ICU = intensive care unit. ^∗^Histogram was constructed to analyze normal distribution of asthma duration. The normal curve displayed that duration of diagnosis was normally distributed.

**Table 2:** Drug utilization among asthmatic patients prior to visiting Armed Forces Referral and Teaching Hospital, Addis Ababa, Ethiopia.

Drugs	Prior to visit *n* (%)	After visit *n* (%)
Monotherapy	85 (46.2)	35 (19.0)
SABA inhaler	45 (24.5)	17 (9.2)
SABA oral	17 (9.2)	5 (2.7)
Oral corticosteroid (OCS)	7 (3.8)	4 (2.2)
Antihistamine	3 (1.6)	3 (1.6)
Parenteral steroid	7 (3.8)	—
Methylxanthine	6 (3.3)	6 (3.3)
Two-drug combination	90 (48.9)	114 (61.9)
ICS+LABA (long-acting beta agonist)	22 (11.9)	21 (11.4)
ICS+SABA inhaler	7 (3.8)	14 (7.6)
OCS+SABA inhaler	6 (3.3)	4 (2.2)
Antihistamine+OCS	7 (3.8)	6 (3.3)
SABA inhaler+SABA oral	39 (21.2)	49 (26.6)
SABA oral+OCS	9 (4.9)	8 (4.3)
Antibiotics+SABA inhaler	—	5 (2.7)
Methylxanthine+SABA inhaler	—	7 (3.8)
Three-drug combination	9 (4.9)	35 (19.1)
SABA inhaler+SABA oral+OCS	9 (4.9)	6 (3.3)
ICS+SABA inhaler+SABA oral	—	7 (3.8)
Antibiotics+OCS+SABA inhaler	—	15 (8.2)
Antibiotics+OCS+SABA oral	—	7 (3.8)

**Table 3:** Univariate logistic regression analysis of asthma control with asthma quality of life at Armed Forces Referral and Teaching Hospital, Addis Ababa, Ethiopia, July-October 2015 (*n* = 184).

Variable	Asthma control		COR (95% CI)	*P* value
	Controlled *n* (%)	Uncontrolled *n* (%)		
Asthma-related quality of life				
Good quality of life	50 (27.2%)	24 (13.0%)	1.00	0.000
Poor quality of life	9 (4.9%)	101 (54.9%)	23.4 (10.12, 54.03)	
Total	59	125	184	
Mean quality of life			6.64 (3.69, 11.95)	0.000

COR = crude odds ratio.

**Table 4:** Multivariable logistic regression analysis of factors associated with poor asthma control among asthmatic patients attending Armed Forces Referral and Teaching Hospital, Addis Ababa, Ethiopia, July-October 2015 (*n* = 184).

Variable	Asthma control		COR (95% CI)	AOR (95% CI)	*P* value
	Controlled *n* (%)	Uncontrolled *n* (%)			
Age category					
18-34	25 (13.6%)	23 (12.5%)	1.00	1.00	
35-64	28 (15.2%)	87 (47.3%)	3.38 (1.7, 6.86)^∗^	6.31 (2.06, 19.3)^∗^	0.001
≥65	6 (3.3%)	15 (8.1%)	2.72 (0.90, 8.2)	1.53 (0.21, 11.23)	0.679
Gender					
Male	28 (15.2%)	76 (41.3%)	1.00	1.00	
Female	31 (16.9%)	49 (26.6%)	0.58 (0.31, 1.09)	0.38 (0.15, 0.98)^∗^	0.044
Marital status					
Single	4 (2.9%)	22 (16.2%)	1.00	1.00	
Married	33 (24.3%)	45 (33.1%)	0.61 (0.27, 1.40)	0.24 (0.08, 0.78)^∗^	0.017
Divorced	2 (1.5%)	9 (6.6%)	2.12 (0.40, 11.3)	1.63 (0.23, 11.43)	0.624
Widowed	4 (2.9%)	17 (12.5%)	1.47 (0.46, 4.69)	0.62 (0.09, 4.43)	0.635
Education level					
No formal education	8 (4.3%)	20 (10.8%)	1.00	1.00	
Primary education	8 (4.3%)	34 (18.8%)	1.70 (0.55, 5.27)	3.39 (0.67, 17.12)	0.140
Secondary education	23 (12.5%)	48 (26.0%)	0.84 (0.32, 2.18)	1.74 (0.48, 6.30)	0.402
Higher institute	20 (10.8%)	23 (12.5%)	0.46 (0.17, 1.27)	0.74 (0.19, 2.94)	0.673
Duration of diagnosis					
≤14.8 years	37 (20.1%)	52 (28.3%)	1.00	1.00	
>14.8 years	22 (11.9%)	73 (39.7%)	2.36 (1.3, 4.46)^∗^	1.33 (0.50, 3.53)	0.562
Comorbid illness					
Yes	9 (4.9%)	56 (30.4%)	1.00	1.00	
No	50 (27.2%)	69 (37.5%)	0.22 (0.1, 0.49)^∗^	0.23 (0.09, 0.61)^∗^	0.003
Hospital admission in the last 12 months					
Yes	5 (2.7%)	34 (18.5%)	1.00	1.00	
No	54 (29.3%)	91 (49.5%)	0.25 (0.1, 0.67)^∗^	0.41 (0.13, 1.27)	0.123
Oral SABA use					
Yes	10 (5.4%)	65 (35.3%)	1.00	1.00	
No	49 (26.7%)	60 (32.6%)	0.19 (0.1, 0.41)^∗^	0.22 (0.09, 0.59)^∗^	0.003
OCS use					
Yes	6 (3.3%)	32 (17.4%)	1.00	1.00	
No	53 (28.8%)	93 (50.5%)	0.33 (0.13, 0.84)^∗^	0.37 (0.11, 1.22)	0.101

^∗^Statistically significant at *P* < 0.05. COR = crude odds ratio; AOR = adjusted odds ratio.

**Table 5:** Multivariable logistic regression analysis of factors associated with poor asthma-related quality of life among asthmatic patients attending Armed Forces Referral and Teaching Hospital, Addis Ababa, Ethiopia, July-October 2015 (*n* = 184).

Variable	Asthma quality of life		COR (95% CI)	AOR (95% CI)	*P* value
	Good QoL *n* (%)	Poor QoL *n* (%)			
Age category					
18-34	27 (14.7%)	21 (11.4%)	1.00	1.00	
35-64	40 (21.7%)	75 (40.7%)	2.41 (1.21, 4.79)^∗^	1.96 (0.71, 5.44)	0.194
≥65	7 (3.8%)	14 (7.6%)	2.57 (0.88, 7.51)	1.55 (0.36, 6.75)	0.557
Gender					
Male	33 (17.9%)	71 (38.6%)	1.00	1.00	
Female	41 (22.3%)	39 (21.2%)	0.44 (0.24, 0.81)^∗^	0.36 (0.16, 0.84)^∗^	0.018
Duration of diagnosis					
≤14.8 years	47 (25.5%)	42 (22.8%)	1.00	1.00	
>14.8 years	27 (14.7%)	68 (37.0%)	2.82 (1.53, 5.19)^∗^	2.18 (0.91, 5.20)	0.079
GINA severity					
Severe persistent	9 (4.9%)	30 (16.3%)	1.00	1.00	
Moderate persistent	12 (6.5%)	42 (22.8%)	1.05 (0.39, 2.81)	1.04 (0.30, 3.63)	0.957
Mild persistent	32 (17.4%)	34 (18.5%)	0.32 (0.13, 0.77)^∗^	0.48 (0.14, 1.69)	0.253
Intermittent	21 (11.4%)	4 (2.2%)	0.06 (0.02, 0.21)^∗^	0.18 (0.04, 0.86)^∗^	0.032
Comorbid illness					
Yes	16 (8.7%)	49 (26.6%)	1.00	1.00	
No	58 (31.5%)	61 (33.2%)	0.34 (0.18, 0.67)^∗^	0.66 (0.24, 1.77)	0.404
Hospital admission in the last 12 months					
Yes	8 (4.3%)	31 (16.8%)	1.00	1.00	
No	66 (35.9%)	79 (42.9%)	0.31 (0.13, 0.72)^∗^	0.53 (0.20, 1.42)	0.203
ICS use					
Yes	23 (12.5%)	6 (3.3%)	1.00	1.00	
No	51 (27.7%)	104 (56.5%)	7.82 (3.0, 20.39)^∗^	1.99 (0.64, 6.24)	0.236
Oral SABA use					
Yes	15 (8.1)	60 (32.6%)	1.00	1.00	
No	59 (32.1%)	50 (27.2%)	0.21 (0.11, 0.42)^∗^	0.39 (0.17, 0.89)^∗^	0.026
OCS use					
Yes	5 (2.7%)	33 (17.9%)	1.00	1.00	
No	69 (37.5%)	77 (41.8%)	0.17 (0.06, 0.46)^∗^	0.22 (0.06, 0.73)^∗^	0.013

^∗^Statistically significant at *P* < 0.05. COR = crude odds ratio; AOR = adjusted odds ratio.

## Data Availability

The derived data used to support the findings of this study are available from the corresponding author upon request.

## Abbreviations

ACT: Asthma Control Test
AFRTH: Armed Forces Referral and Teaching Hospital
AQLQ: Asthma Quality of Life Questionnaire
COPD: Chronic obstructive pulmonary disease
GINA: Global Initiative for Asthma
HRQoL: Health-related quality of life
ICS: Inhalation corticosteroids
LABA: Long-acting beta agonists
OCS: Oral corticosteroids
QoL: Quality of life
SABA: Short-acting beta agonist.

## Additional Points

*Limitations of the Study*. Absence of objective measurement (spirometer or peak flow meter) of airflow obstruction and lung function test might have led to misdiagnosis of some patients, such as smokers (<5%), as asthmatic instead of COPD. However, the inclusion criteria ruled out this possibility. The effect of smoking on asthma control and QoL was not studied, as the number of smokers was very small. Some comorbidities such as rhinitis and other conditions like family history of asthma and environmental conditions could also influence the result.
